# Identification of the Bony Canal of the Posterior Superior Alveolar Nerve and Artery in the Maxillary Sinus: Tomographic, Radiographic, and Macroscopic Analyses

**DOI:** 10.1155/2015/878205

**Published:** 2015-03-16

**Authors:** Iris Jasmin Santos German, Daniela Vieira Buchaim, Jesus Carlos Andreo, Elio Hitoshi Shinohara, Ana Lúcia Alvares Capelozza, Andre Luis Shinohara, Geraldo Marco Rosa Junior, Mizael Pereira, Rogerio Leone Buchaim

**Affiliations:** ^1^Department of Biological Sciences (Anatomy), Bauru School of Dentistry, University of São Paulo (FOB-USP), Al. Dr. Octávio Pinheiro Brisola 9-75, 17012-901 Bauru, SP, Brazil; ^2^University of Marilia (UNIMAR), Medical School, Discipline of Human Morphophysiology, Rua Hygino Muzy Filho, 17525-902 Marília, SP, Brazil; ^3^Israeli Albert Einstein Hospital, Maxillofacial Surgery Service, Avenida Albert Einstein 627, Morumbi, 05652-900 São Paulo, SP, Brazil; ^4^Department of Stomatology, Discipline of Radiology and Stomatology, Bauru School of Dentistry, University of São Paulo (FOB-USP), Bauru, SP, Brazil; ^5^University of Sacred Heart, Rua Irmã Arminda, 10-50 Jardim Brasil, 17011-160 Bauru, SP, Brazil

## Abstract

The aim of this study was to identify the shape and route of the bony canal of the posterior superior alveolar artery (PSAA) and posterior superior alveolar nerve (PSAN) using different identification methods, including computed tomography (CT), panoramic radiograph, and macroscopic evaluation (corpse and dry skull). Twenty-four patients were analyzed by CT and panoramic and posterior anterior (PA) radiographs; additionally, 90 dry skulls and 21 dissected anatomical specimens were examined. Three-dimensional-CT revealed that the lateral wall of the maxillary sinus resembled a tunnel format in 60% of the treated patients. Out of all 24 patients, the panoramic radiograph identified the bony canal in only one patient; whereas the PA radiograph identified it in 80% of the patients. The dry skulls showed tunnellike routes of the PSAA and PSAN in 65% of the cases. Moreover, the pathway was also visibly observed in the dissected anatomical specimens as a straight shape in 85% of the cases. Thus, our results demonstrated that the most common shape of the bony canal of the PSAA and PSAN is the tunnel format with a straight route by 3D-CT, posterior anterior radiography, and macroscopic evaluation. However, in the panoramic radiographs, it was difficult to identify this canal.

## 1. Introduction

The vascular system of the maxillary sinus varies in architecture and vascular anastomosis of the vessels and involves the presence of the infraorbital artery, the anterior superior alveolar artery (ASAA), and the posterior superior alveolar artery (PSAA) [1, 2].

The infraorbital artery originates in the pterygopalatine fossa accompanied by the infraorbital nerve; it emerges from the inferior orbital fissure, leaving the pterygopalatine arteries and giving rise to the ASAA on the passage through the infraorbital canal [3–5]. This arterial branch should be identified by imaging studies before surgery, as it is commonly located between the lower and middle thirds of the anterior sinus wall and thereby frequently located over the bone window required for the establishment of grafts and the installation of implants [6].

The PSAA, a branch of the maxillary artery, passes through the pterygomaxillary fissure. It meets the posterior superior alveolar nerve (PSAN) and accompanies it through the alveolar foramen in the alveolar maxillary tuberosity and the infratemporal fossa [3]. The PSAN occupies the slender foramina in the lateral and posterior walls of the maxillary sinus, joins the PSAA, and passes down adjacent to the maxillary tuberosity [4].

The PSAA and PSAN cross a canal in the bony wall of the maxillary sinus, whose location has been previously described in scientific papers through various methodologies such as computed tomography (CT) studies on cadavers [7–10] and anatomical specimens [11, 12]. This canal is classified into three types: canal, groove-shaped (tunnel-like), and fragmented (intraosseous and extraosseous route) [13]. In a previous study, the presence of this canal was detected on CT scans in 53% of the cases evaluated, at a distance of more than 16 mm from the alveolar crest in 80% of radiographs [7]. In addition, CT scans from patients undergoing maxillary sinus augmentation procedures showed that the PSAA appeared intraosseous in 68.2% of cases [8].

The direction of the infraorbital artery and the PSAA has been classified into two categories, namely, type 1 (straight) and type 2 (“U” shaped). In type 2, changes of direction occur either in the region between the first and second premolar or between the second premolar and the first molar [14].

The distance from the bottom edge of the PSAA to the bone crest has obtained approximate mean values of length from different authors: 18.9–19.6 mm [12]; 18.90 ± 4.21 mm and 15.45 ± 4.04 mm, in the premolar and molar regions, respectively [15]; 16 ± 3.5 mm [7]; 16.9 mm [9]; and 17.03 mm [16]. However, this distance becomes relatively meaningless because the loss of bone crest drastically alters these numbers. The distance of the bony canal from the crest differs depending on the crestal bone loss, the location in the maxillary, and the presence or absence of teeth [7, 12, 17, 18].

Based on these previous data, and due the current lack of studies evaluating the presence of this bony canal, this study aimed to assess the bony canal of the neurovascular bundle of the PSAA and PSAN through different methodologies (CT, panoramic radiographs, posterior anterior radiographs, and evaluation of dry skulls and dissected anatomical specimens) in order to elucidate the best assessment method of this anatomical structure.

## 2. Materials and Methods

Twenty-four patients were analyzed by panoramic radiographs, cone beam CT, and posterior anterior radiographs (equal sex distribution); in addition, 90 dry skulls (mean age 60 years and equal sex distribution) and 21 dissected anatomical specimens were also analyzed. Images were captured using an SLR digital camera (Nikon Coolpix L810 16.1 MP 26x Optical Zoom, Nikon FM2, Nikon Corporation, Tokyo).

### 2.1. Computed Tomography Imaging

For the evaluation of the maxillary sinus, we used the I-CAT CT scanner (Cone Beam Volumetric Tomography (I-CAT Vision Program) and Imaging Sciences International (Kavo, Moema, São Paulo, Brazil)). Each image and anatomic structure was assessed thoroughly in order to identify the course of the bony canal related to the adjacent structures, in addition to its disposal extraosseous and intraosseous routes.

**Figure 1:**
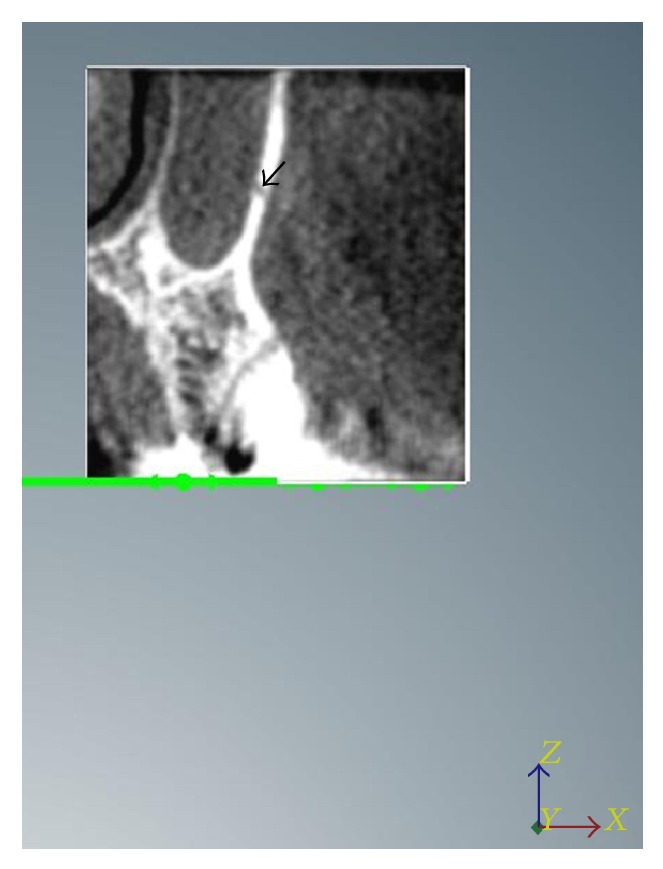
Coronal reconstruction showing the route of the bony canal (black arrow).

**Figure 2:**
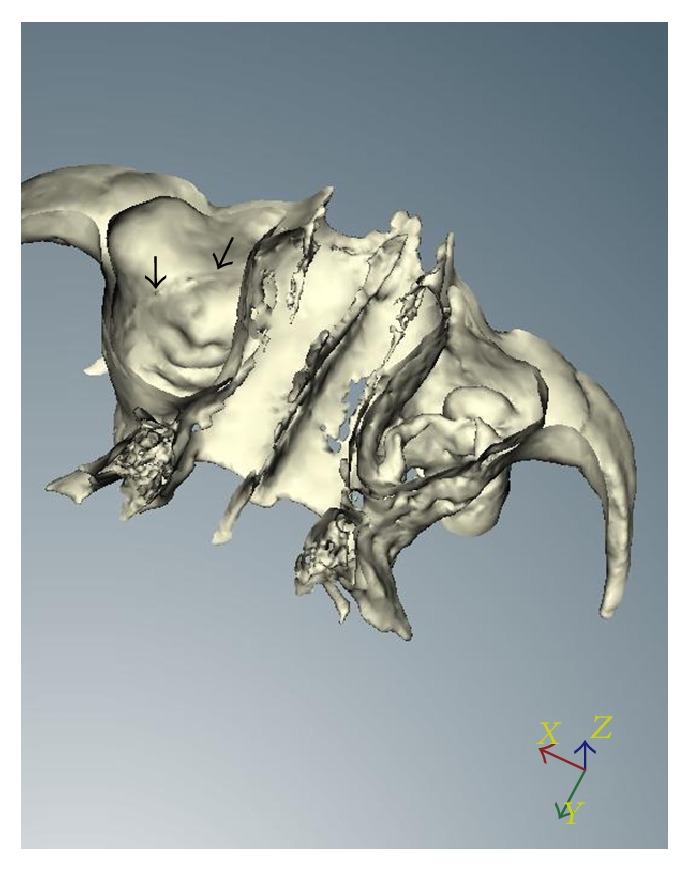
Three-dimensional reconstruction of the bony canal (black arrows).

Aiming to reduce the movements of the device, the patients were positioned according to the protocol and monitored during an exposure time of 40 seconds. The parameters of the I-CAT CT scanner showed the following specifications: the images were scanned at 120 kVp and 3–7 mA, using a focal point of 0.5 mm, voxel size of 0.2 mm, and 14 BITs (grayscale) FOV (field of view) at the standard diameter of 16 cm diameter × 13 cm height (maximum). Images were obtained through the program Xoran CAT Software (Xoran Technologies, Ann Arbor, USA) (Figures 1-2). For 3D reconstruction, Nobel Clinician Software (Nobel Biocare, Kloten, Switzerland) was used.

### 2.2. Panoramic Radiograph

**Figure 3:**
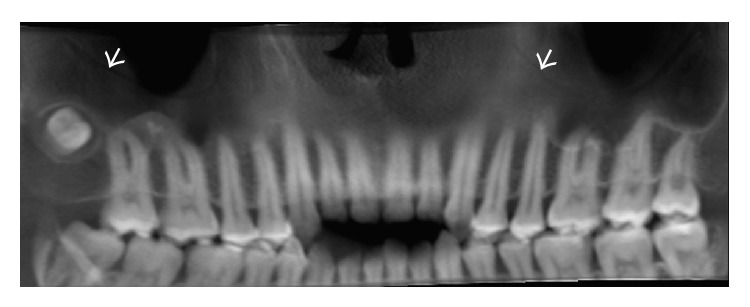
Panoramic radiograph of the bony canal pathway (white arrows).

The Panoramic Rotograph Plus (Villa Sistemi Medicali, Buccinasco, MI, Italy) was used for the panoramic radiograph. The exposure time was 17 seconds (75 kV) at intervals of 240 s exposures (1 : 16 duty cycle) and a focus-patient distance of 150 cm. The patient was monitored according to the manufacturer's protocol.  Figure 3 exhibits the pathway of the PSAA and PSAN in the panoramic radiograph (white arrows).

### 2.3. Posteroanterior (PA) Radiograph

**Figure 4:**
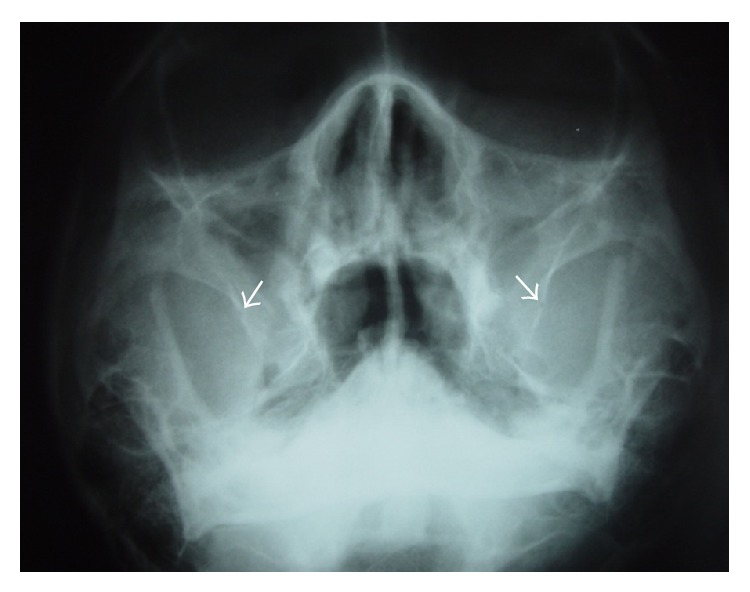
Posteroanterior radiograph of the maxillary sinus (Water's projection) showing a radiolucent area corresponding to the route of the neurovascular bundle (white arrows).

The PA radiographs were taken in the PA view using the Rotograph Plus, with ear rods placed into both the external auditory meatus of the patients and the face turned to superior and posterior direction (Figure 4).

### 2.4. Macroscopic Evaluation: Cadaver Dry skull

**Figure 5:**
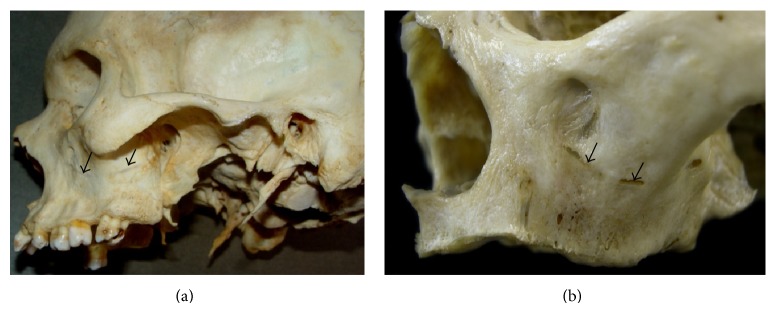
Route of the bony canal in the dry skulls (black arrows). (a) A tunnel route; (b) an extraosseous and intraosseous route (fragmented route).

Ninety dry skulls were obtained from the Department of Basic Sciences, Faculty of Dentistry of Araçatuba of São Paulo State University (UNESP), Brazil. The skulls were chosen according to the optimal identification and topographical distributions of the bony canals. Figures 5(a) and 5(b) show the route of the PSAA and PSAN in the dry skulls (black arrows).

### 2.5. Macroscopic Evaluation: Dissected Anatomical Specimens

**Figure 6:**
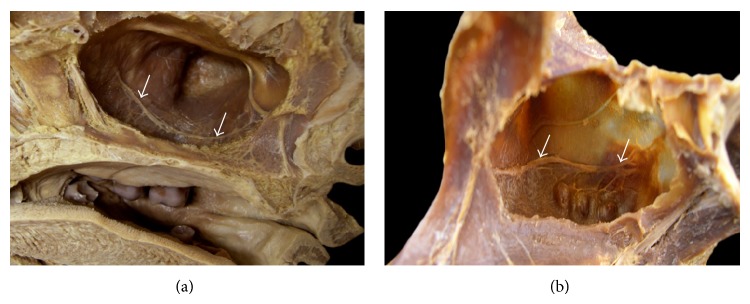
(a, b) Route of the posterior superior alveolar neurovascular bundles in dissected anatomical specimens (white arrows).

Twenty-one dissected anatomical specimens were analyzed, and the PSAA and PSAN were identified by examining the internal face of the maxillary sinus. These anatomical pieces were obtained from the Department of Basic Sciences, Faculty of Dentistry of Araçatuba of São Paulo State University (UNESP), Brazil. The specimens were dissected with the assistance of a magnifying visor (3x magnification; ESTEK). The dissection procedure consisted of opening, cutting in the median sagittal plane, removal of the nasal septum and nasal concha to gain access to the maxillary sinus, and posterior dissection of the lateral wall of the maxillary sinus. Figures 6(a) and 6(b) show the route of the PSAA and PSAN in the dissected anatomical specimens (internal view of the lateral walls of the maxillary sinuses, white arrows).

## 3. Results

The methods used in this study were able to identify the bony canal. The 3D-CT revealed that the lateral wall of the maxillary sinus appeared as a tunnel shape in 60% of the treated patients (58% and 62% on the right and left side, resp.), showing a bone impression of the PSAA and PSAN (Figures 1-2).

Out of all 24 patients, the panoramic radiograph was able to identify the bony canal in only one patient.

The PA radiograph showed a radiopaque area corresponding to the route of the PSAA and PSAN. This bony canal was found in 80% of patients (Figure 4).

Analysis of dry skulls revealed that the route of the neurovascular displayed a tunnel-like shape in 65% of cases, while it was found to be fragmented in 20% of the cases. These different bony canal routes are shown in Figure 5.

Moreover, the pathway of the PSAA and PSAN was also visibly observed in the dissected anatomical specimens. A total of 15% of the dissected specimens showed a “U” shaped feature (Figure 6(a)), whereas the remaining 85% of the specimens showed a “straight” shape (Figure 6(b)).

## 4. Discussion

To the best of our knowledge, this is the first study identifying the bony canal of the PSAA and PSAN neurovascular bundle using several different methods, especially using panoramic radiographs.

As mentioned, in this study, we identify the route and localization of the neurovascular bundle through different methodologies. This anatomical region has a clinical importance because of the vascularity of the maxillary sinus and its implication during trauma. In the coronal reconstruction, the PSAA and PSAN appeared intraosseous, which is the most common type according to Güncü et al. [8].

Three-dimensional reconstruction of the bony canal showed a straight format (type 1), according to the classification of Hur et al. [14], with changes of direction occurring in the regions between the first and second premolar or between the second premolar and the first molar.

From the 21 dissected anatomical specimens analyzed in this study, we found both directions of the PSAA; however, the straight shape was found in 85%, whereas a “U” shape was found in only 15%, as classified by Hur et al. [14].

In the panoramic radiographs, the bony canal was observed in only one patient. This result is interesting because the identification of this bone canal by radiographic analysis is uncommon. The use of a panoramic radiograph for this purpose is unacceptable and is below the “standard of care.” Our finding was accidental and probably reflects an artery route of over 3 mm in diameter, which is rare but does occur. Accordingly, the panoramic radiograph is not a reliable diagnostic tool to identify this bony canal, owing to its low accuracy compared with other diagnostic imaging methods, such as CT scans [19].

Regarding the intraosseous anastomosis between the PSAA and the infraorbital artery, there are some controversies in the current literature. This anastomosis was found in 80% of the CT scans analyzed in the studies by Traxler et al. [17] and Rosano et al. [11]. However, in the studies by Elian et al. [7] and Mardinger et al. [9], the presence of this artery was only detected in approximately 50% of CT images. Traxler et al. [17] stated that there is an artery anastomosis in 47.25% and 44.5% of cases among the PSAA and infraorbital artery in in both intraosseous and extraosseous pathways, respectively, with a 1 mm diameter of the canal. Moreover, it has been shown that the PSAA and infraorbital artery branch appeared separately from the maxillary artery in 42.9% of 21 analyzed cadavers [20].

Rosano et al. [18] found an intraosseous anastomosis between the PSAA and infraorbital artery in 100% of cases in their study using dissected human cadavers. The CT scan revealed 3 courses: a completely intraosseous course in 100% of cases; a partially intraosseous route, especially from the second premolar to second molar; and a variable course (intraosseous or subperiostal or intrasinusal) in the area of the maxillary tuberosity. In our study, CT revealed a tunnel format (intraosseous course) in 60% of cases. This result is similar to that identified by Elian et al. [7] and Kim et al. [15], where an intraosseous route was detected in 51.4% of cases on the right side and in 54.3% on the left side (average 52.9%). Further, this result is fairly similar to the findings of Temmerman et al. [19], who found an intraosseous canal strongly evident in 49.5% of the cases.

The dry skulls examined herein most frequently exhibited a tunnel route, followed by the fragmented shape. As reported by Sato et al. [13], this canal can be classified as three types, namely, canal (15%), tunnel (68%), and fragmented shapes (17%).

Understanding of this anatomical area is important not only to avoid threats to the neurovascular bundle, but also to avoid mechanical damages. The anastomosis of the arteries of the maxillary sinus and its location can enhance the risks of vascular trauma or interference with graft angiogenesis during surgery for maxillary sinus augmentation [10, 21, 22] and, hence, may produce heavy bleeding during surgical procedures [23–25]. Surgical planning for augmentation of the maxillary sinus also involves analyzing the correlation between the dimensions of the bone window and the formation of vital bone. A lower risk of stroke and increased bone formation were observed in cases with smaller bone window in a previous study [26]. Due to the proximity of nerve fibers (PSAN) and PSAA, there is a high risk of ischemia, pain, and/or inflammation during surgical treatments in the area [13].

The involvement of the PSAA in injuries during the treatment of Le Fort I fractures, as diagnosed by digital angiography, has been previously described [27]. Osteotomy of the lateral wall of the maxillary sinus can cause damage to the PSAA, leading to bleeding, which is characterized by obvious nasal bleeding [28, 29]. This artery can also be damaged during other surgical procedures comprising this anatomical area, including maxillary sinus augmentation, removal of pathologic lesions and infections in the maxillary sinus, orthognathic surgery, and dental implant placement. Nerve block resulting from an incorrect injection technique of the extraosseous route of the PSAA and PSAN can lead to hematoma development secondary to vascular invasion of the needle [30]. To avoid these accidents, it becomes necessary to identify the PSAA and PSAN by preoperative CT [28]. Moreover, it has been reported that a safe area for the injection is located in a triangle location near the upper maxillary second molar, which minimizes the risk of hematomas [30].

In this study, it was possible to identify the bony canal corresponding to the PSAA and PSAN by tomographic, radiographic, and macroscopic analyses. Anatomical knowledge of this bony canal would be highly valuable for medical professionals and dentists, particularly in the field of periodontics, implantology, and maxillofacial surgery, guiding them to pursue the proper care of the region of the maxillary artery and its branches. Consequently, possible complications resulting from damage to the vascular system of this anatomical region, especially in fragile bone types with alveolar resorption, would be minimized.

However, more researches are still needed in order to identify this bony canal by different evaluation methods, especially in relation to the possible anatomical changes, and according to the presence or absence of teeth, in order to ensure greater safety during surgical procedures involving the maxillary sinus and its associated vascularization.

## 5. Conclusions

Our results demonstrated that the most common shape of the bony canal of the PSAA and PSAN is the tunnel shape with a straight route by 3D-CT and posterior anterior radiography and macroscopic evaluation. However, this canal was not easily identified upon panoramic radiographs.

The PSAA may be damaged during surgical procedures, including surgeries for Le Fort I fractures, maxillary sinus augmentation, removal of pathologic lesions and infections in the maxillary sinus, orthognathic surgery, and dental implant placement, causing severe bleeding. Further, incorrect blocking of the extraosseous route of PSAN during anesthesia may lead to hematoma by vascular invasion of the needle, and the identification and diagnosis of this artery and nerve by CT could minimize the occurrence of bleeding and nerve damage. The identification of this bony canal provides a broader view of the neurovascular bundle, thus minimizing the risk of potential complications during clinical procedures in this anatomic area.
